# Application of Lung Ultrasound in Critical Care Setting: A Review

**DOI:** 10.7759/cureus.5233

**Published:** 2019-07-25

**Authors:** Ronak Raheja, Megha Brahmavar, Dhruv Joshi, Dileep Raman

**Affiliations:** 1 Internal Medicine, Kempegowda Institute of Medical Sciences, Bengaluru, IND; 2 Internal Medicine, Cloudphysician Healthcare, Bengaluru, IND

**Keywords:** thoracic ultrasound, intensive care, blue protocol, ultrasound modes, artifacts

## Abstract

This article reviews the use of thoracic ultrasound in the intensive care unit (ICU). The focus of this article is to review the basic terminology and clinical applications of thoracic ultrasound. The diagnostic approach to a breathless patient, the blue protocol, is presented in a simplified flow chart. The diagnostic application of thoracic ultrasound in lung parenchymal and pleural diseases, role in bedside procedures, diaphragmatic assessment, and lung recruitment are described. Recent updates discussed in this review help support its increasingly indispensable role in the emergent and critical care setting.

## Introduction and background

The introduction of lung ultrasound has revolutionized the care of patients in a modern ICU. It has also shown an impact in non-ICU settings such as in pulmonology and thoracic surgery ambulatory clinics [1]. Historically, lung ultrasonography (LUS) has been a neglected area given perceived notions about the utility of this modality in air-filled structures. However, in the last two decades, significant progress has been made in using ultrasonography as a valuable tool in evaluating lung pathologies.

Aim

We aim to review the application of lung ultrasound in bedside clinical medicine and introduce it as an adjunct to the stethoscope in physical examination. We review the basic points required to introduce lung ultrasound to physicians. We aim to simplify the approach to a breathless patient by creating a simplified flowchart of the blue protocol which helps the reader quickly arrive at the cause of breathlessness using an ultrasound probe. We also aim to review the utility and application of lung ultrasound in pleural and parenchymal lung pathologies and also cover the use of ultrasound in thoracic procedures. 

Advantages of lung ultrasound

Lung ultrasonography has many advantages that are immediately recognizable, and a few have been listed below:

1. Cost: Ultrasonography is relatively inexpensive, and has almost no consumable costs.

2. Safety: It does not utilize ionizing radiation. It can be used safely in pregnant women.

3. Repeatability: It is safely repeatable at the patient’s bedside for the countless number of times to monitor progress and response to treatment.

4. Efficiency: It efficiently gives immediate information to healthcare providers. In an emergency setting, radiography may be of suboptimal value to take immediate and efficient decisions, an attempt to calculate the efficiency of chest radiography to deliver adequate timely information during daily rounds in an intensive care setting showed it to provide the required information only 63% of the time [2]. Lung ultrasound can provide a quicker and more efficient means of providing instantaneous information at the bedside.

5. Adjunct to physical examination: A study by D. Lichtenstein et al demonstrated that chest auscultation when performed alone was able to successfully detect 61% of pleural effusions, 36% of consolidations, and 55% of alveolar-interstitial syndromes [3]. Lung ultrasound has been shown as a powerful adjunct to physical examination. 

6. Decreases iatrogenic complications: Rahman et al. demonstrate that physicians trained in ultrasonography perform ultrasound-guided invasive procedures with outcome rates that are at par with those performed by interventional radiologists [4]. This indicates a need to have more physicians trained in lung ultrasound. The inability to use ultrasound in performing invasive critical procedures subjects patients to preventable harm.

7. Decreased transfer of critically ill patients: Oks et al. demonstrate that the overall cost and time benefit is better for ultrasound as compared to transferring critically ill patients for computerized tomography (CT) scans [5].

Minimum requirements to practice lung ultrasound

A person must at least meet the basic skill requirements as stated by the American College of Chest Physicians/La Société de Reanimation de Langue Française statement or British Thoracic Society pleural disease guideline 2010 or equivalent to be considered competent to perform critical care ultrasound [6-7].

Disadvantages

Ultrasound is operator dependent and the quality of images may vary depending on the technique and skill which requires a steep learning curve. Interobserver variability makes it difficult to replicate ultrasound studies and make generalizable conclusions on its utility. 

Hardware

*Basic Settings on Ultrasound Machine: *Various settings such as gain, time compensation, and depth have been described by Willamson et al and are useful in obtaining a clear and well-balanced image [8]. For lung ultrasound, the probe is placed in the intercostal space. This provides adequate penetration to visualize the pleura and lungs. The ribs are radiopaque and do not provide a good medium for visualization. Linear probes may be used to provide a better definition of the pleura and to more clearly visualize some of the signs below.

There are three basic modes to study ultrasound.

*Amplitude Mode (A-Mode)*: Amplitude mode is the display of amplitude spikes on the screen with X (depth) and Y (amplitude) axis plotted as a graph. It is frequently used to study the eye and in pin-point procedures like lithotripsy [9].

 

*Motion Mode (M-Mode)*: A sequence of two dimensions with time on the horizontal axis and tissue depth on vertical axis follow each other and help in the detection of movement of organ boundaries as the depth from the probe changes in moving organs. This has good applications in the emergency setting to diagnose pneumothorax, left ventricular systolic function, cardiac tamponade, and hypertrophic obstructive cardiomyopathy [10]. 

 

Brightness mode (B-mode): (mode of choice unless mentioned otherwise): An array of transducers produce a planar 2D image. This is the most commonly used mode and is the mode of choice in our discussion unless mentioned otherwise [9].

The hardware of ultrasound machines is constantly evolving, from older and larger bedside machines to small convenient pocket-sized machines. The choice of a probe is based on how much tissue penetration is desired as shown in Table 1. Each probe is designed to emit a set of frequencies ranging from 1 MHz to 15 MHz. The resolution of an image is better at higher frequencies, but this comes at the expense of depth of penetration. The higher frequency probes are used to visualize superficial structures, like chest wall masses and have a tissue penetration of 3-6 cm with good image resolution. The convex probes are used over soft surfaces like the abdomen to obtain a tissue penetration of up to 15-20 cm but at a lower resolution. Usually, a convex 3.5-5 MHz probe is used for lung ultrasound as shown in Table 1.

**Table 1:** Choice of frequency and probe type for ultrasound Abbreviations: FAST: focused assessment with sonography for trauma, DVT: deep vein thrombosis

Frequency	Use
2.5 MHz to 5 MHz Curvilinear	Thoracic ultrasound Blue protocol, thoracentesis, abdominal aortic aneurysm, deep abdomen ultrasound, obstetric, and gynecological ultrasound FAST scan
3.5 to 5.0 MHz Phased array	Echocardiogram and lung ultrasound, Thoracentesis, FAST
6 MHz to 11 Linear array	Breast, thyroid, carotids, retinal scans, musculoskeletal, DVT, pleural masses, vascular ultrasound
12 to 15 MHz B scan probe	Eyeball

## Review

Approach to performing lung ultrasound 

**Figure 1:**
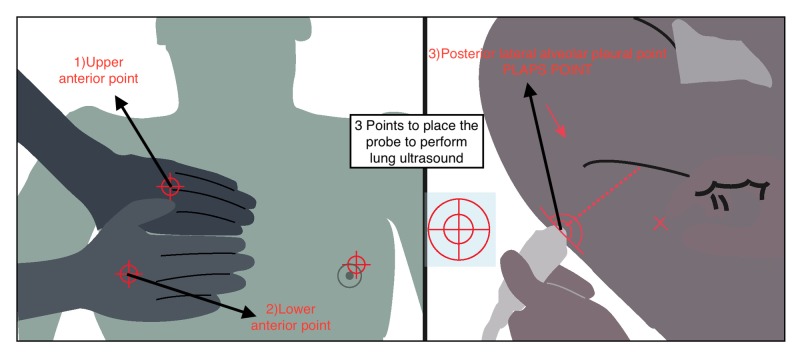
Three basic points where lung ultrasound should be performed

The approach to performing lung ultrasound is systematic and step-wise. The ultrasound probe is primarily placed on the three described points as shown in Figure 1. 

Normal lung ultrasound picture

**Figure 2:**
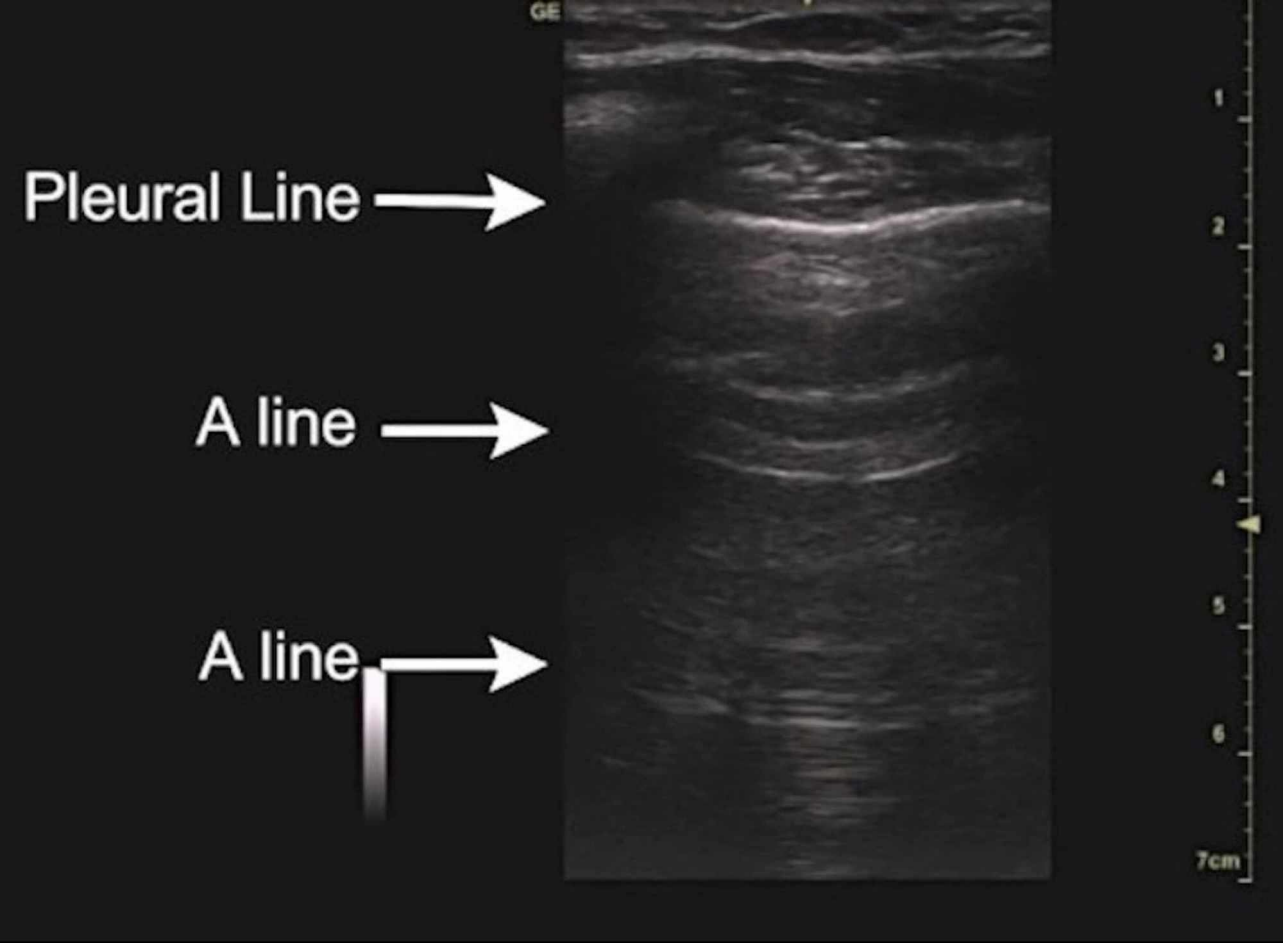
A lines are an important component of the normal lung ultrasound profile

The normal lung profile corresponds to the A profile as illustrated in Figure 2 in accordance with the blue protocol. The A profile is when we see A lines with normal lung sliding. A lines are shown in Figure 2.

On the ultrasound image, the equidistant reflections of the pleural line in the air parenchyma of the lung are called A lines [8]. It is important to understand that the lung ultrasound picture may be normal (A profile) in the case of asthma, chronic obstructive pulmonary disease, acute exacerbation of COPD, and pulmonary embolism as described in the blue protocol below. 

Utility of ultrasound in acute dyspnea.The Blue protocol 

**Figure 3:**
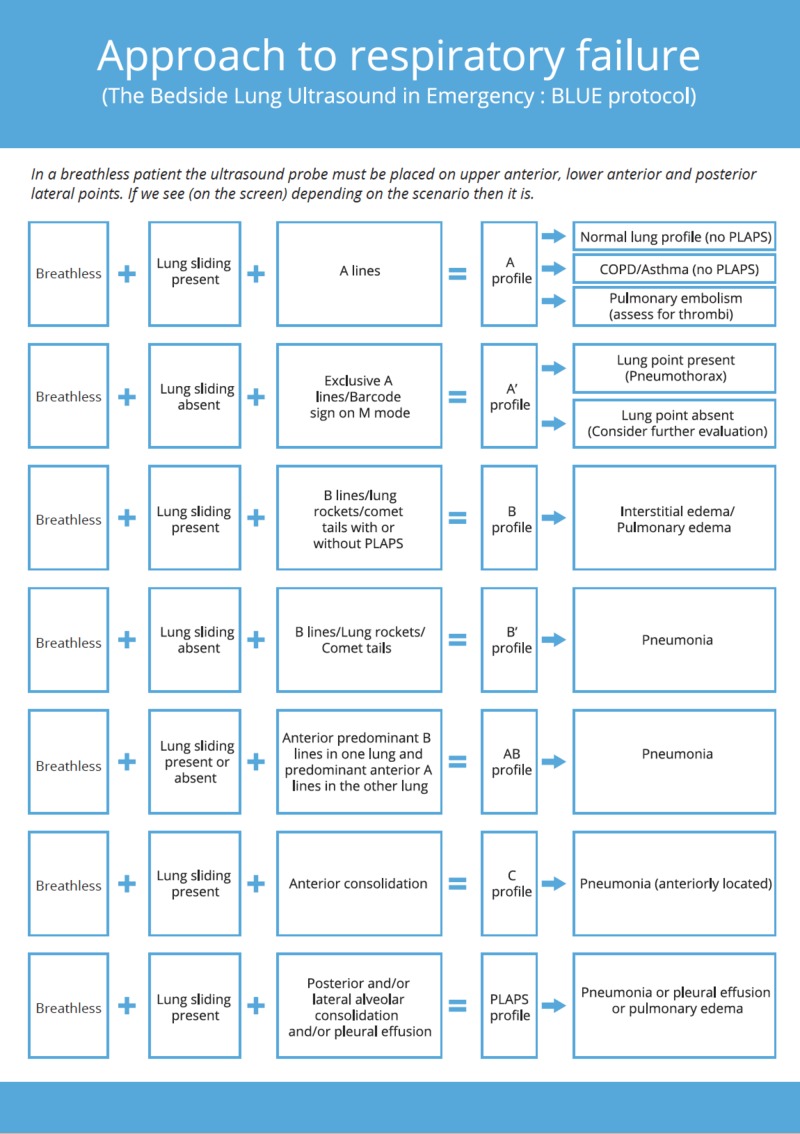
The Blue protocol

We can arrive at the cause of dyspnea by using the Blue protocol, simplified and structured in Figure 3. It demonstrates the utility of ultrasonography as a powerful adjunct to physical examination in the evaluation of the cause of breathlessness [11]. It is important to take into consideration that in Figure 3 the first prerequisite is that the patient is breathless. In a breathless patient, the ultrasound probe is placed in the described points. A detailed explanation of the various lung artifacts can be found in the respective sections of the article.

Utility of ultrasound in alveolar exudates and pulmonary edema 

**Figure 4:**
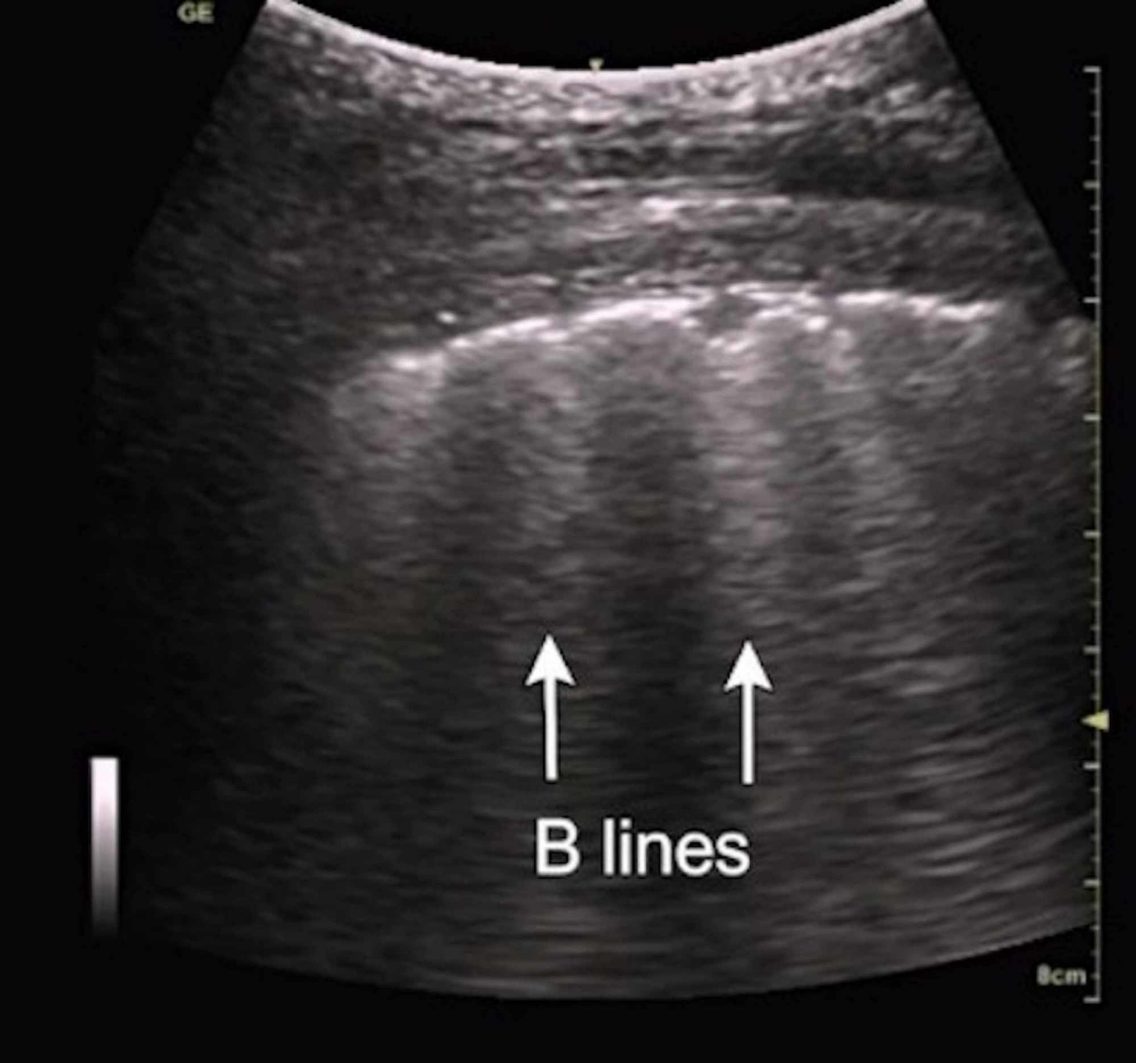
B lines

Pulmonary edema and exudates are usually best diagnosed with the help of B lines and PLAPS profile. B lines shown in Figure 4 are produced when one object has a large difference in acoustic impedance as compared to the surrounding structures. These appear as vertical artifacts projecting from the pleural line to the bottom of the screen known as lung rockets or comet tails, these are referred to as B lines. As the lung parenchyma is mainly composed of air these may be produced by interstitial fluid, edema in pulmonary edema or ARDS or even solids with different acoustic impedance from air-like collagen scarring/fibrosis [12]. The density of B lines may help in the prediction of the severity of pulmonary edema [8].

**Figure 5:**
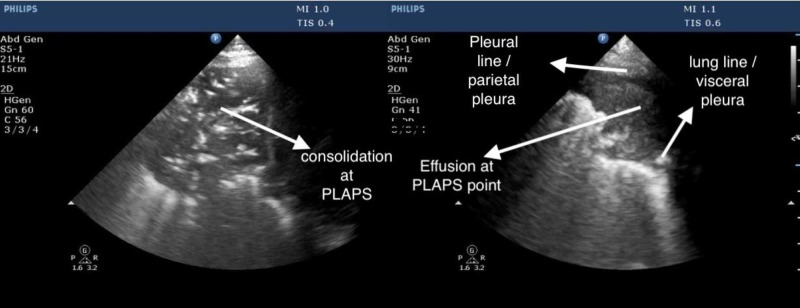
Posterior lateral alveolar and pleural point (PLAPS)

Posterior lateral alveolar and the pleural point is a posterior and lateral point on the chest which shows an early collection of fluid or exudates in the dependent regions of the thorax [11]. In the case of pneumonia, effusion, or pulmonary edema the fluid generally flows to the posterior lateral alveolar and pleural point as PLAPS point shown in Figure 5, which is gravity dependent. These have been combined together to form a PLAPS profile in the Blue protocol.

Utility of ultrasound in pneumonia

Pneumonia may be diagnosed if the consolidation is in continuity with the pleural membrane. On placement of the ultrasound probe in the upper anterior, lower anterior, or PLAPS points, a solidified liver tissue like image usually appears on the screen. The presence of dynamic air bronchograms assists in the diagnosis of pneumonia. It is critical for the person performing the scan to identify and demarcate the liver with the help of the diaphragmatic line of separation to avoid confusion with pneumonia. The diaphragm is highly reflective, and the liver diaphragm surface can be reflected in the air of normal lung tissue as an artifact, which may be misinterpreted as hepatization of the lower lobe of the lung. Chest CT is the gold standard for diagnosis of pneumonia, however, it has a high radiation exposure and high cost. Chest radiographs are used most frequently in clinical practice but have a poor sensitivity of 43.5% for the diagnosis of pneumonia (95% CI, 36.4%-50.8%) [13]. Nazerian et al. demonstrate lung ultrasound as superior to chest X-ray for diagnosing pneumonia in the ICU setting [14]. Lung ultrasound is reliable, rapid, and conclusive to arrive at a diagnosis of pneumonia even in the emergency room [15].

Utility of ultrasound in atelectasis

The immediate changes in ultrasound are the absence of lung sliding and a still cupola with lung pulse which are signs of poor lung expansion. The cupola (or cervical pleura) is the continuation of the costal and mediastinal parts of the pleura over the apex of the lung. Lung pulse is a phenomenon where the static pleural line moves due to the reverberations of the beating heart [16]. Sensitivity and specificity of lung pulse to detect complete atelectasis is 93% and 100% [17]. Atelectasis can be seen in 10%-50% of postoperative patients, depending on the type of surgery [18]. As mentioned above in addition to absent lung sliding and still cupola atelectasis may appear like alveolar consolidation but with absent dynamic air bronchograms. Even if air bronchograms are present secondary to trapped air in the bronchi, they are usually static in the case of atelectasis [16]. There will also likely be signs of loss of lung volume which is indicated by the heart sign. The heart sign indicates that the heart is displaced secondary to loss of lung volume and it can be visualized anywhere in the right or left chest. The ultrasound findings for atelectasis may be present even before radiological findings are seen [19].

Utility of ultrasound in lung abscess

A lung abscess is a thick walled collection of pus within the lung. A lung abscess appears as a hypoechoic mass with an anechoic central portion with or without septae. A mildly hyperechoic peripheral wall may also be seen [20]. Lung ultrasound can detect 94% of lung abscesses. A 94% success rate is seen on the US-guided aspiration of the detected abscesses with a 6% risk of developing pneumothorax [21]. Ultrasound targeted therapy of administration of antibiotics inside the abscess was shown to decrease the duration of systemic antibiotics needed to cause a resolution of the abscess [22].

Utility of ultrasound in pneumothorax

The diagnosis of pneumothorax requires a sequential examination and presence of signs as noted below in a defined order as in the Blue protocol. No single sign should be taken in isolation for optimal sensitivity and specificity.

**Figure 6:**
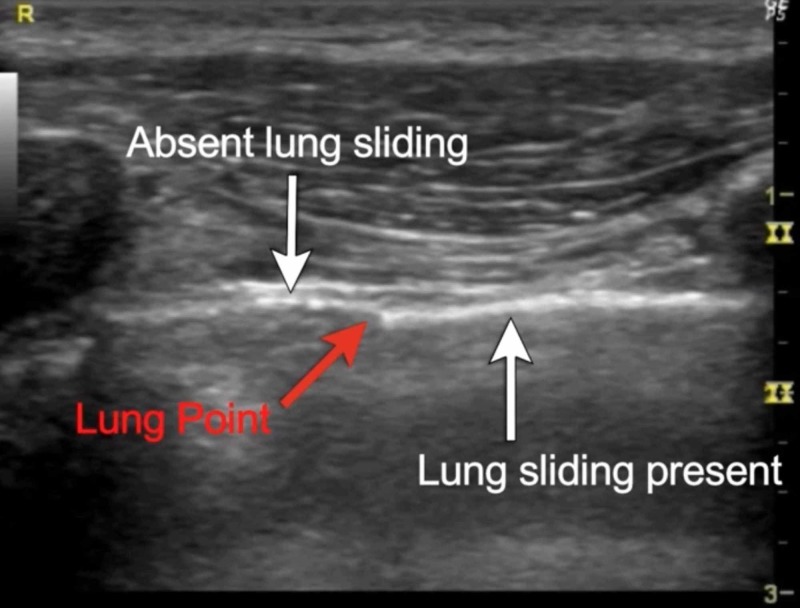
Lung point

**Figure 7:**
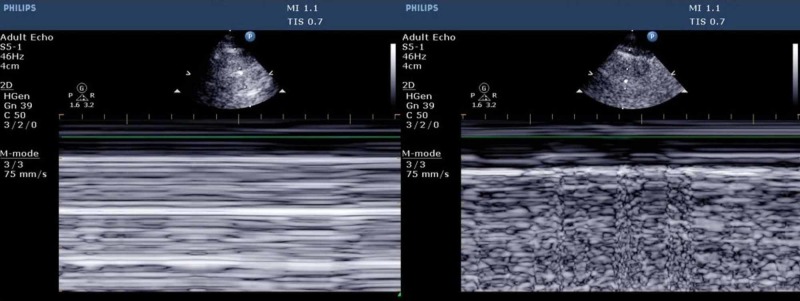
Barcode sign and seashore sign

1. Lung sliding must be absent to suspect pneumothorax at the point of examination.

2. An A-line pattern must also be seen on B mode. On the M mode the seashore sign is replaced by the barcode sign as seen in Figure 7.

3. Lung point: The lung point (Figure 6) is a transition point where lung sliding disappears. This point is the location where parietal and visceral pleura part due to the appearance of air between them. This should be the last sign that should be looked for after the above two in the same sequence.

As loss of lung sliding can also be seen in chronic obstructive pulmonary disease and pleural adhesions, it is important to use all three signs in the correct order to diagnose pneumothorax [23]. Absent lung sliding at a point, followed by the demonstration of the lung point in Figure 6 has a sensitivity of 95.3%, specificity of 91.1% to predict the presence of a pneumothorax. The presence of lung sliding has a negative predictive value of 100% for pneumothorax at that location [24].

The presence of B lines indicates the absence of pneumothorax at that position, however, B lines can be absent in normal lung profiles and may not be very reliable for making a diagnosis [25]. Ultrasound may be the best method to diagnose traumatic pneumothorax and iatrogenic pneumothoraxes at bedside [26]. The results of lung ultrasound are more sensitive than supine chest radiographs for pneumothorax [27-28]. Ultrasound has a sensitivity of 78%-90% in diagnosing traumatic pneumothorax in comparison to 39%-52% with chest radiographs [29]. The distance from the mid axillary line to lung point can be useful in estimating the size of pneumothorax [30]. A drawback of lung ultrasound is the inability of ultrasound to pick up pneumothorax in cases of subcutaneous emphysema or in presence of chest wall bandages [30]. The normal lung on ultrasound shows lung sliding with granular moving artifacts which appear like seashores on M mode [24]. In the absence of lung sliding on M mode, the seashore sign is lost and appears like a fixed barcode as shown in Figure 7. 

Utility of ultrasound in pleural effusion

**Figure 8:**
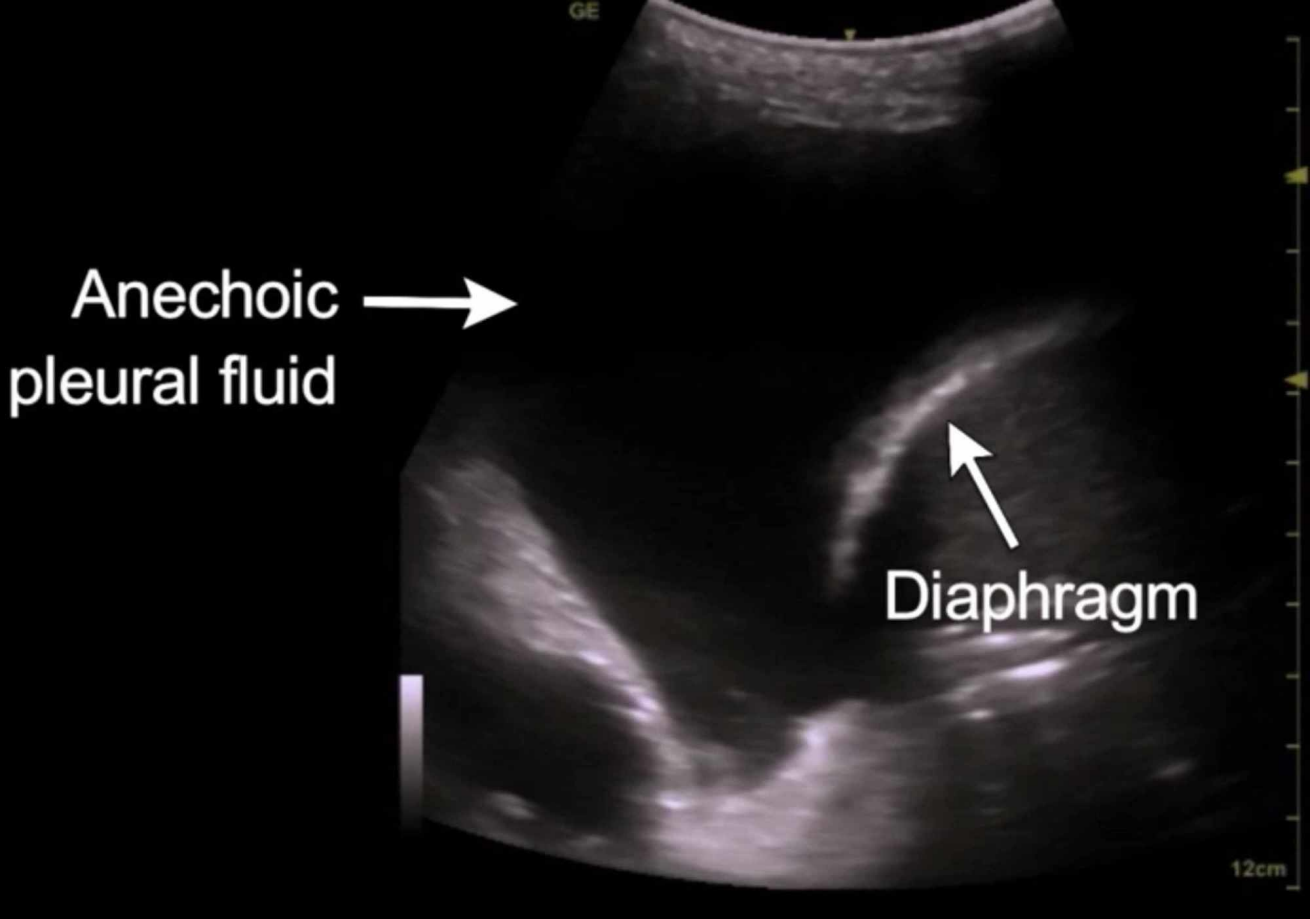
Quad sign in pleural effusion

Pleural effusion can be diagnosed on ultrasonography reliably by the quad sign seen in Figure 8. The quad sign is bound by parietal pleura above and visceral pleura below and by rib shadows on either side [31]. The visceral pleura oscillates with respiration giving rise to the sinusoid sign on the M mode [31]. Ultrasound of pleura has a sensitivity of 93% in detecting pleural effusion when compared to radiographs that have a sensitivity of 83%, the absence of fluid was determined in 89% of cases in ultrasound as compared to chest x-ray which could detect absence of fluid in 61% of cases [32]. The PLAPS point is one of the first points where we can pick up effusions as fluid collection is gravity driven [33].

Ultrasonography can help estimate the amount of effusion by the formula V(ml) = 20 × Separation between two pleura (mm). Mean prediction error of V was 158.4 ± 160.6 ml to estimate the volume of fluid in between pleura [34]. We can predict the type of effusion based on echogenicity and classify it as either a transudate or exudate. Transudative pleural effusions are usually anechoic whereas exudates maybe echoic or anechoic [8]. When combined with the above, a pleural thickening of more than 1 cm, pleural nodularity, or echogenic swirling can help in suspecting a malignancy [35].

Ultrasound in chest wall masses and neoplasms

Lung ultrasound may be effective, convenient, and economical to assess the size and location of a chest wall mass and help in the biopsy of the mass lesion [20]. Neoplasms of the lung can only be seen provided they abut the pleura. Chest wall invasion is suggested if there is an interruption in the pleural line of pleural sliding signs [36]. Tumors of the chest wall appear as well-defined and hypoechoic masses between the soft tissue layers. In contrast, inflammatory lesions look irregular with a heterogeneous echotexture. Bone invasion by a chest wall lesion has hyperechoic plate-like shadows within the lesion. Osteolytic bone lesions are hypoechoic with an outer ring shadow. Malignant lymph nodes are round, hypoechoic, and single or multiple confluent and lobulated masses [37]. According to Chira et al., ultrasound was better at identifying intra-tumoral necrosis in 87.1% patients as compared to CT which could identify intra-tumoral necrosis in 72.8% of patients [38]. Another study by Bandi et al showed ultrasound was more sensitive (89%) for the assessment of chest wall involvement as compared to CT scans (42%) [39]. Ultrasound has adequate potential in identifying dangerous intercostal and percutaneous blood vessels while performing biopsies of chest wall masses [40]. The value of ultrasound has been underestimated in the evaluation of chest wall masses and tumor invasion. CT may have a higher sensitivity and specificity in detecting neoplasms overall, but ultrasonography may prove to be a useful adjunct in some settings. Further studies are needed to evaluate the role of ultrasound in chest wall masses.

Utility of lung ultrasound in thoracic procedures

One of the important uses of thoracic ultrasound is in the insertion of subclavian central lines and the confirmation of their position. This also reduces the need for a confirmatory x-ray, thus hastening the administration of drugs and reducing healthcare costs [41]. It also reduces the rate of complications like pneumothorax, hemothorax, venous tear, arterial puncture, hematoma, and nerve injury [42]. The pleural avoidance with rib trajectory (PART) technique has been tested as a means of reducing pneumothorax risk with central line placement [43]. Mechanical complications using the “blind” technique are as high as 18.8% in subclavian line insertions. Research suggests that the infraclavicular, longitudinal “in-plane” technique is the preferred method for subclavian line placement as it is the only view that allows for a direct ultrasonographic view of needle visualization [42]. Ultrasound is especially useful in locating peripheral lung lesions where bronchoscopy is not accessible [44]. Lung ultrasound showed improved outcomes in successful tapping with a decreased rate of complications when compared to blind tapping [33]. Lung ultrasound may be useful in monitoring and evaluation of the resolution of pneumothorax after chest tube placement [45].

Utility of lung ultrasound in diaphragmatic dysfunction.

Chest radiography can be used to detect diaphragmatic paralysis with a sensitivity of 90%, but a specificity of only 44% [46]. The other methods to identify diaphragmatic dysfunction are sniff test, pulmonary function tests, trans-diaphragmatic pressure (Pdi), ultrasonography, and electromyography. The diaphragm will normally contract and thicken during inspiration while the chronically paralyzed diaphragm is atrophied and will be thinner. Ultrasonography measures the thickness of the diaphragm at the zone of apposition (i.e., the region where the diaphragm abuts the lower rib cage) during inspiration. A paralyzed diaphragm can be identified by the combination of a diaphragm thickness of less than 2 mm at functional residual capacity and a change in thickness (during inspiration) of less than 20% [47]. Ultrasound of the diaphragm can also be used in patients to assess weaning off the ventilator. The change in the thickness of the diaphragm (tdi) using Δtdi% (percent change in tdi between end-expiration and end-inspiration) is directly related to the success of extubation in patients during liberation trials. If the change in thickness of the diaphragm at the end of inspiration is more than 30%, then the success rate for liberation is high [48]. 

Utility of lung ultrasound in PEEP titration.

Ultrasound can be used in recruitment maneuvers and PEEP (Positive End Expiratory Pressure) titration as well. Bedside ultrasound can detect lung collapse which helps in selecting patients for recruitment maneuvers. Moderate, severe and complete loss of lung aeration is represented on ultrasound by the presence of multiple B-line (B1 lines), coalescent B-lines (B2 lines) and consolidation [49]. Inspiratory pressures are obtained by identifying the pressure required for the image to shift from a consolidated lung to a normal lung. The minimum PEEP required to prevent lung collapse is the pressure recorded when there is a shift from the normal lung image to a B1-B2 pattern plus 2 cm water [50].

## Conclusions

The role of lung ultrasound in the ICU is evolving. Even though its utility is not in question, one of the shortcomings of ultrasound has always been that it is operator dependent. It is therefore hard to extrapolate results from experienced centers to other areas. The value of ultrasound must be proven in diverse clinical settings by operators of varying backgrounds to lend credibility to its broad use. The levels of statistical certainty (which are high in some studies) stated in this review will need further refinement to consolidate the evidence base in the favor of lung ultrasound. Adequate training and a good understanding of sonographic properties of the thorax are essential to building expertise in bedside lung ultrasound. There is a need to include a structured training program for physicians during their training years. We are cautiously optimistic about the role ultrasound will play in tomorrow’s ICUs and emergency rooms.
